# Supplementary material for: The interactome of intact mitochondria by cross-linking mass spectrometry provides evidence for coexisting respiratory supercomplexes
Source: Mol Cell Proteomics. 2017 Dec 8;17(2):216–32. doi: 10.1074/mcp.RA117.000470 (PMC5795388; doi:10.1074/mcp.RA117.000470)
Supplement: Supplemental Data [file supp_RA117.000470_133922_0_supp_23978_fzffwf.zip › spectra_annotation/mito_spectra_annotation/329-1-6-1-10-1.pdf]

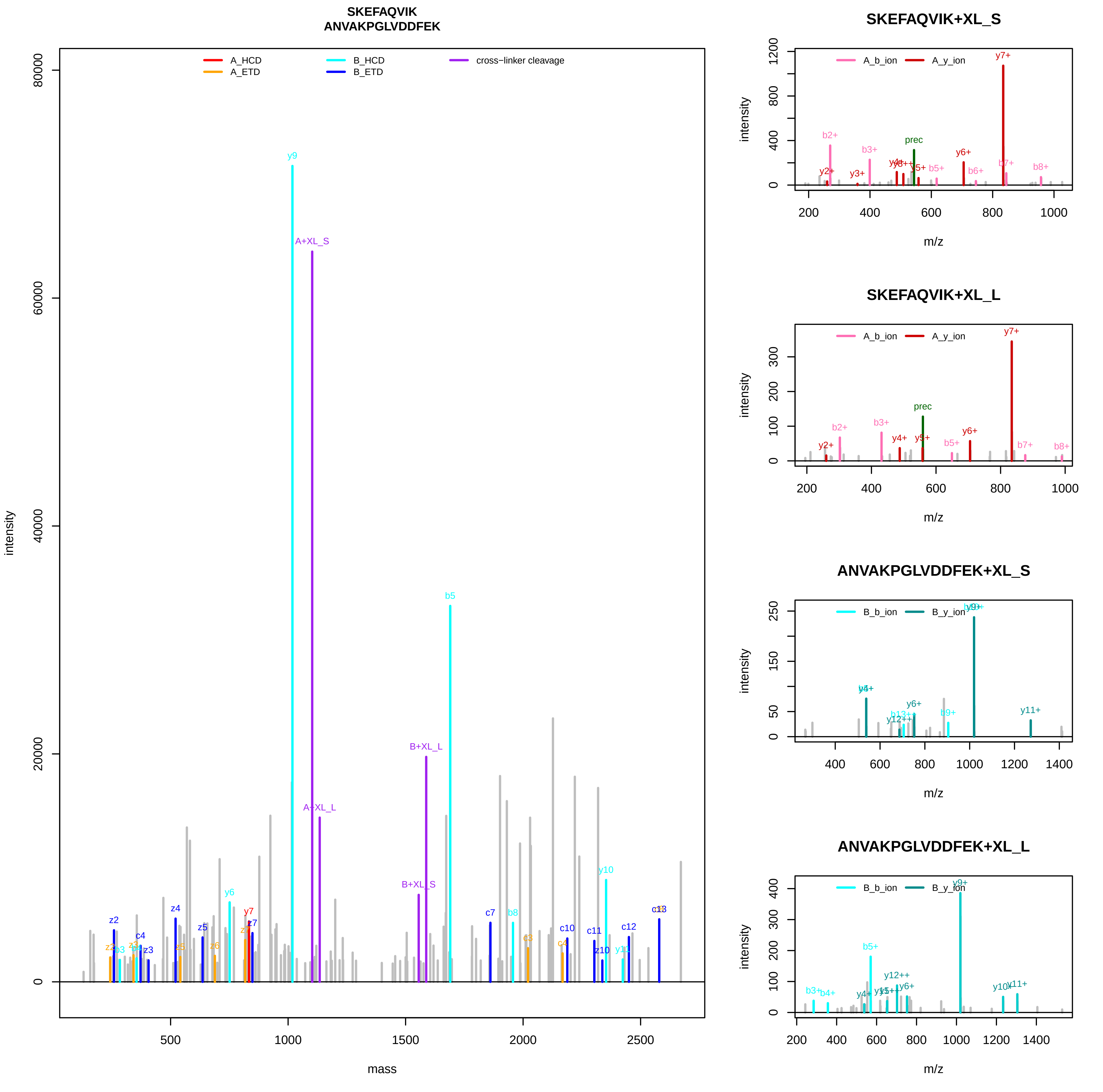
SKEFAQVIK
ANVAKPGLVDDFEK
A_HCD
A_ETD
B_HCD
B_ETD
cross−linker cleavage
intensity
mass
0
20000
40000
60000
80000
500
1000
1500
2000
2500
y9
A+XL_S
A+XL_L
B+XL_L
B+XL_S
b5
y6
y7
z7
z2
z4
z5
z6
c4
z3
c7
b8
c10
c11
z10
y10
y11
c12
c13
SKEFAQVIK+XL_S
A_b_ion
A_y_ion
m/z
y7+
prec
b2+
b3+
y2+
y3+
y6+
b5+
b6+
b7+
b8+
y5+
SKEFAQVIK+XL_L
y4+
ANVAKPGLVDDFEK+XL_S
B_b_ion
B_y_ion
y9+
y6+
b9+
y11+
y12++
ANVAKPGLVDDFEK+XL_L
b3+
b4+
b5+
y12++
y10+
